# Discrimination and reliability: Equal partners?

**DOI:** 10.1186/1477-7525-6-81

**Published:** 2008-10-16

**Authors:** Geoffrey R Norman

**Affiliations:** 1 McMaster University, Hamilton, Canada

## Abstract

A critique of Hankins, M article: How discriminating are discriminative instruments?" *Health and Quality of Life Outcomes *2008, **6**:36

## Letter to the editor

There are several definitions of discrimination. Two, from the Webster dictionary, are: 1) the process by which two stimuli differing in some aspect are responded to differently, and 2) the quality or power of finely distinguishing. It seems to me that the manuscript by Hankins [1], in attempting to elaborate on 1), shows considerable absence of 2).

To begin with, a small disclaimer: The “McMaster Framework” is hardly endorsed by all at McMaster. In fact, my co-author Dave Streiner and I, both of us originally from McMaster, in our textbook [2] specifically challenge the Kirshner and Guyatt [3] notion of different kinds of instruments for different purposes.

And now to the matter at hand. Hankins [1] attempts to show that reliability is not a good measure of discrimination, and that instruments can be reliable but not discriminating, and vice versa. But, while he refers to formulas for both reliability and discrimination (more on this in a moment), he does not actually define either. This is not just pedantry; in my view, reliability is, by definition, an index of the ability of an instrument to discriminate among individuals. To quote an authority on the subject, me [1]:

“..the reliability coefficients reflects the extent to which a measurement instrument can differentiate among individuals, *since the magnitude of the coefficient is directly related to the variability between subjects”*.

An almost perfect paraphrase of the Webster definition above. Fundamentally *all* reliability coefficients are intraclass coefficients, and mathematically reflect the proportion of the variance in the observations that relate to real differences among subjects. The formula is:

$$\text{Reliability}=\frac{\text{Variance}\left(\text{Subjects}\right)}{\text{Variance}\left(\text{Subjects}\right)+\text{ Variance }\left(\text{error}\right)}$$

The numerator expresses variability due to different responses among individuals. The denominator expresses all variability. So reliability is a measure of the extent to which people differ, expressed as a number between 0 and 1. QED – reliability *is* discrimination. This is also precisely consistent with Hawkins' “test discrimination”. However there is a very important concept buried in the formula. Discrimination and reliability is a matter of *true* differences between subjects, in contrast to differences arising from measurement error. Simply finding difference among observed scores is insufficient, since these may arise either from true differences or error. True differences can only be separated from measurement error by conducting a study where there are multiple observations on each subject.

Hankins[1] presents a series of examples where the measures of reliability and discrimination (Ferguson's Delta) diverge. Let us look at example 1, where two scales are administered to 10 subjects. See Table 1 for the data.

**Table 1:** Data used by Hankins's to illustrate the calculation of delta: where two scales are administered to 10 subjects.

Subject	A	B	C	D	E	F	G	H	I	J
Response I	1	2	2	3	3	3	3	4	4	5

Response II	1	1	1	1	1	1	1	1	3	5

Average	1.0	1.5	1.5	2.0	2.0	2.0	2.0	2.5	3.5	5.0

According to Hankins, the ICC relating I to II is 0.83, so the reliability is high. (Actually, by my calculations it's 0.704). This comes about because, to estimate the reliability, we use both sets of observations to estimate the average score for each individual, then calculate the variance due to differences between subjects (true variance) equal to 1.11, and the residual or error variance, equal to 0.467. The ratio of 1.11 to (1.11+0.47) is 0.70. However, Hankins' point is that Scale B is less discriminating since there are far more ties, and he shows this by computing Ferguson's delta for I = 0.925, and for II = 0.425. One conceptual problem is immediately apparent. In the calculation of reliability, one needs to treat I and II as repeated observations of the same scale, in order to estimate true and error variance. So it makes no sense to then turn around and talk about whether Scale I is better or worse at discriminating than Scale II.

Indeed, in talking separately about Scale I and II, Hankins makes the implicit assumption that all the differences within each scale are real; i.e. arise from true differences among the subjects. But this is a heroic assumption, and in fact makes the whole issue of reliability/discrimination tautological. If the differences arise from real differences, then the reliability and discrimination is 1.0 for both scales, since there is no measurement error. But there is no way, in a sequence of observations, one per subject, to decide whether these differences are true or error. The numbers shown above could have arisen simply from rolling a die in front of each person and writing down the number that came up. Both sequences could arise plausibly from the toss of a die. Surely it makes no sense to discuss whether one random sequence of die tosses is more discriminating than another.

Another way of looking at it. Since the names, A, B, → J, are completely arbitrary, I could rearrange one sequence with no loss of information. Suppose now the data looks like this (see Table 2).

**Table 2:** Data from table 1 with one sequence rearranged.

Subject	A	B	C	D	E	F	G	H	I	J
Response I	1	2	2	3	3	3	3	4	4	5

Response II	5	3	1	1	1	1	1	1	1	1

Average	3.0	2.5	1.5	2.0	2.0	2.0	2.0	2.5	2.5	3.0

According to Hankins, I continues to be more discriminating than II, since it's the same numbers. The delta for I remains .925, and for II it is .425. However, this time the reliability is zero, since the between subject variance is zero (The estimate is negative, but negative variances cannot exist). The discrimination of Scale A and B are irrelevant. The fact that the two scales are unrelated (inversely related) belies any claim of good discrimination.

Let's try another example. Look at the set of numbers in Table 3.

**Table 3:** Data used to illustrate the flaw in Hankins's argument that Scale II is more discriminating than Scale I.

Observation	Weight I	Weight II
1	100	100.057

2	100	100.817

3	100	100.045

4	100	100.678

5	100	100.233

6	100	100.566

7	100	100.344

8	100	100.287

9	100	100.456

10	100	100.653

Well, according to Hankins, Scale I is useless, since all observations are the same and Delta is 0. Scale II is terrific, since all observations are different, and Delta = 1. Trouble is these (admittedly fictitious) data all came about from me repeatedly stepping on the scales this morning, using an old-fashioned analog scale that could only be read to +/- 1 kg, and a fancy new digital scale, with readout (but not accuracy) good to +/- 1 gm.

Surely we cannot advance the argument that Scale II is more discriminating than scale I, since all observations are of the same person and all variation is error.

If you want to look at reliability or discrimination, you must separate error variance – differences between individual observations and the average for each individual, from true variance-differences between individuals. It requires more than one observation to do that. But unless you can meaningfully separate signal from noise, you cannot look at the precision of measurement, whether you call it reliability, discrimination, or anything else.

Delta does not require a second administration. You can get it all with one shot. But you cannot get something for nothing. The problem with Delta is that all it cares about are differences. These differences may come about because of error variance or true variance. Unless the two are separated, nonsensical conclusions result.

Obscure coefficients are like obscure composers; there's likely a good reason why they have faded into obscurity.

## Competing interests

The author declares that he has no competing interests.

## References

[B1] Hankins M (2008). How discriminating are discriminative instruments?. Health and quality of life outcomes.

[B2] Streiner D, Norman G (2003). Health Measurement Scales – A practical guide to their development and use.

[B3] Kirshner B, Guyatt G (1985). A methodological framework for assessing health indices. Journal of chronic diseases.

